# The OsHAPL1-DTH8-Hd1 complex functions as the transcription regulator to repress heading date in rice

**DOI:** 10.1093/jxb/erw468

**Published:** 2017-01-02

**Authors:** Shanshan Zhu, Jiachang Wang, Maohong Cai, Huan Zhang, Fuqing Wu, Yang Xu, Chaonan Li, Zhijun Cheng, Xin Zhang, Xiuping Guo, Peike Sheng, Mingming Wu, Jiulin Wang, Cailin Lei, Jie Wang, Zhichao Zhao, Chuanyin Wu, Haiyang Wang, Jianmin Wan

**Affiliations:** 1 National Key Facility for Crop Gene Resources and Genetic Improvement, Institute of Crop Science, Chinese Academy of Agricultural Sciences, Beijing, PR China; 2 National Key Laboratory for Crop Genetics and Germplasm Enhancement, Nanjing Agricultural University, Nanjing, PR China

**Keywords:** DTH8, Ehd1, HAP, Hd1, Hd3a, heading date, *Oryza sativa*, OsHAPL1, RFT1

## Abstract

Heading date is an important agronomic trait related to crop yield. Many genes related to heading date have already been identified in rice (*Oryza sativa*), and a complicated, preliminary regulatory genetic network has also already been established, but the protein regulatory network is poorly understood. We have identified a novel heading date regulator, Heme Activator Protein like 1 (OsHAPL1), which inhibits flowering under long-day conditions. OsHAPL1 is a nuclear-localized protein that is highly expressed in leaves in a rhythmic manner. OsHAPL1 can physically interact with Days To Heading on chromosome 8 (DTH8), which physically interacts with Heading date 1 (Hd1) both *in vitro* and *in vivo*. OsHAPL1 forms a complex with DTH8 and Hd1 in *Escherichia coli*. OsHAPL1, DTH8, and Hd1 physically interact with the HAP complex, and also with general transcription factors in yeast (*Saccharomyces cerevisiae*). Further studies showed that OsHAPL1 represses the expression of the florigen genes and *FLOWERING LOCUS T 1* (*RFT1*) and *Hd3a* through *Early heading date 1* (*Ehd1*). We propose that OsHAPL1 functions as a transcriptional regulator and, together with DTH8, Hd1, the HAP complex, and general transcription factors, regulates the expression of target genes and then affects heading date by influencing the expression of *Hd3a* and *RFT1* through *Ehd1*.

## Teaser

Flowering time is an important trait for determining the adaptability of a crop cultivar. A novel heading date regulator, OsHAPL1, interacts with DTH8-Hd1 to negatively regulate flowering time in rice.

## Introduction

Flowering (or heading in cereals) involves the transition from vegetative to reproductive growth in plants. The correct flowering time is not only important for reproductive success, but also affects crop quality and grain yield (Simpson and Dean, 2002). Multiple internal and external signals, including photoperiod, temperature, and phytohormonal levels, are involved in the flowering process (Simpson and Dean, 2002; Andrés and Coupland, 2012).

Photoperiod is the most important environmental signal determining flowering time. Previous studies have already identified many photoperiod-related genes and the regulatory genetic network of flowering in the model plants *Arabidopsis* and rice (*Oryza sativa*). In Arabidopsis (a long-day (LD) plant), the *CONSTANS* (*CO*) gene is regulated by *GIGANTEA* (*GI*, a circadian clock gene) (Park *et al.*, 1999; Huq *et al.*, 2000), and activates expression of *FLOWERING LOCUS T* (*FT*, a florigen gene in Arabidopsis) (Kardailsky *et al.*, 1999). The FT protein moves to the shoot apical meristem and activates the expression of floral-determination genes to trigger flowering (Wigge *et al.*, 2005; Corbesier *et al.*, 2007). In rice (a short-day (SD) plant), flowering is controlled by an activation pathway in SD and a suppression pathway in LD conditions. *Heading date1* (*Hd1*, the *CO* homologue in rice) is regulated by *OsGI* (the *GI* homologue in rice) (Yano *et al.*, 2000; Hayama *et al.*, 2003), and *Hd1* promotes flowering by activating *Hd3a* expression under SD conditions and delays flowering by repressing *Hd3a* expression under LD conditions (Yano *et al.*, 2000; Kojima *et al.*, 2002; Komiya *et al.*, 2008). The mechanisms are unknown. In addition, rice has a unique *Early heading date 1* (*Ehd1*) pathway that is independent of the *Hd1* pathway (Doi *et al.*, 2004). *Ehd1*, which has no homologue in *Arabidopsis*, encodes a B-type response regulator that promotes the expression of the florigen genes and *FLOWERING LOCUS T 1* (*RFT1*) and *Hd3a* (Doi *et al.*, 2004). Many genes regulating the expression of *Ehd1* have been identified. OsCOL4 (Lee *et al.*, 2010), Grain number, plant height and heading date 7 (Ghd7) (Xue *et al.*, 2008), Days To Heading on chromosome 8 (DTH8/Ghd8/OsHAP3H) (Xue *et al.*, 2008; Wei *et al.*, 2010), and OsCOL10 (Tan *et al.*, 2016) function as repressors of *Ehd1*. OsMADS51 (Kim *et al.*, 2007), Rice Indeterminate 1 (RID1)/Early heading date 2 (Ehd2) (Matsubara *et al.*, 2008; Wu *et al.*, 2008), Early heading date 3 (Ehd3), and Early heading date 4 (Ehd4) (Matsubara *et al.*, 2011; Gao *et al.*, 2013) function as positive regulators of *Ehd1*. Interestingly, a recent study has shown that a MYB-type protein, TaMYB72 from wheat (*Triticum aestivum*), a LD crop, promotes flowering under LD conditions when expressed in rice, suggesting functional conservation in flowering time control between the two diverse crops (Zhang *et al.*, 2016).

mRNA is transcribed by RNA polymerase II, which is controlled by DNA elements in promoters and enhancers recognized by trans-acting factors and general transcription factors (GTFs; Tjian and Maniatis, 1994). Trans-acting factors regulate gene expression in a developmentally or tissue-specific cell-cycle or stimuli-dependent manner, whereas GTFs regulate all promoters as a whole multi-subunit holoenzyme (Parvin and Young, 1998). Some elements are present in most promoters, such as CCAAT boxes in animals, fungi, and plants (Bucher, 1990; Maity and de Crombrugghe, 1998; Mantovani, 1999). Previous studies have shown that CCAAT-binding proteins are NF-Y subunits composed of three parts: NF-YA (CBF-B, HAP2 in yeast), NF-YB (CBF-A, HAP3), and NF-YC (CBF-C, HAP5) (McNabb *et al.*, 1995; Sinha *et al.*, 1995; Mantovani, 1999). In rice, there are 11 *HAP2* genes, 12 *HAP3* genes, and 12 *HAP5* genes (Thirumurugan *et al.*, 2008; Li *et al.*, 2016), each containing a conserved domain and non-conserved regions of varying length and amino acid sequence. The conserved domain is responsible for DNA binding and protein–protein interactions (Thirumurugan *et al.*, 2008). Genes of each family member have different expression patterns, and there are various combinations of overlapping expression of HAP2, HAP3, and HAP5 (Thirumurugan *et al.*, 2008). The biological functions of some HAP family members have recently been identified. High expression of OsHAP3E can induce a dwarf statured plant with erect leaves, whereas overexpression of OsHAP2E confers resistance to pathogens, salinity, and drought, and can increase photosynthesis rate and tiller number (Ito *et al.*, 2011; Alam *et al.*, 2015). OsBF-YC2 and OsNF-YC4 inhibit flowering in rice under LD conditions (Kim *et al.*, 2016), whereas OsHAP5A, OsHAP5B, OsHAP3E, and OsHAP3D delay heading in LD conditions (Li *et al.*, 2016). Although some HAP family members relating to heading date have been identified, the possible complexes involved in heading date are poorly understood.

Our group has been investigating the mechanisms controlling heading date by overexpressing hundreds of transcription factors in rice, and we have identified *OsCOL10* and *OsCOL13* as negative regulators (Sheng *et al.*, 2016; Tan *et al.*, 2016;). Here, we identify a new rice heading date regulator that encodes a histone-like transcription factor, Heme Activator Protein like 1 (OsHAPL1). OsHAPL1 downregulates expression of the florigen genes *Hd3a* and *RFT1* via *Ehd1*, possibly by interacting with Hd1, the HAP complex, and GTFs. Our findings demonstrate a role of OsHAPL1 as a negative heading date regulator, thus providing insight into the flowering control network in rice.

## Materials and methods

### Plant materials and growth conditions

Transgenic plants were produced in the *O. sativa* japonica cv. Kita-ake background. Growth conditions included natural long days (NLD; Beijing, 40°13’N, 116°13’E), natural short days (NSD; Hainan, 18°48’N, 110°02’E), controlled long days (CLD; in growth chambers, 14 h light at 30°C/10 h darkness at 25°C), and controlled short days (CSD; in growth chambers, 10 h light at 30°C /14 h darkness at 25°C). *ehd1*, *hd3a*, and *rft1* mutants in Nipponbare were created using the CRISPR-*Cas9* genome editing system.

### Vector construction

The *Pro*_*Ubi*_*::OsHAPL1-VP64* plasmid was produced using the *OsHAPL1* coding sequence (CDS) driven by the maize ubiquitin (Ubi) promoter fused to the binary vector LP042 nVP64-hyg-asRED via the Gateway cloning system. The *Pro*_*Ubi*_*::OsHAPL1-Flag* plasmid was produced using the *OsHAPL1* CDS driven by the maize Ubi promoter fused to the binary vector pCAMBIA1390 via an In-Fusion Advantage PCR Cloning Kit (Clontech). The *Pro*_*OsHAPL1*_*::Genome*_*OsHAPL1*_*-GFP* plasmid was produced from the genomic DNA sequence and driven by the *OsHAPL1* promoter fused to the binary vector pCAMBIA1390 using the In-Fusion Advantage PCR Cloning Kit. The *oshapl1* mutant was obtained using the CRISPR-Cas9 system. The *Pro*_*35S*_*::OsHAPL1-GFP* plasmid was produced using the *OsHAPL1* CDS driven by the cauliflower mosaic virus (CaMV 35S) promoter fused to the pAN580 vector. Vectors for yeast two-hybrid assays were constructed by CDS of relevant genes fused to pGBK T7 or pGAD T7 (Clontech). Vectors for pull-down assays were obtained by fusing gene CDS to the vectors pGEX-2T(GST), pET-28a(+)(His), and pMAL-c2X(MBP). Vectors for bimolecular fluorescence complementation (BiFC) were constructed by fusing the gene CDS to pSPYNE173 or pSPYCE(M) (Kerppola, 2006). All primers used in the study are listed in Supplementary Table S1, available at *JXB* online.

### Quantitative real-time reverse transcription PCR

Total RNA was extracted using an RNA Prep Pure Kit (Zymo Research, Orange, CA, USA). A reverse transcription (RT) kit (Qiagen) was used in RT reactions. Quantitative RT-PCR (qRT-PCR) was performed using SYBR Premix Ex Taq Kit (TaKaRa; RR041A) in an ABI PRISM 7900HT (Applied Biosystems), and ubiquitin was used as the internal control. Standard errors were calculated from three biological replicates.

### Subcellular localization of OsHAPL1 protein

*Pro*_*35S*_ *::OsHAPL1-GFP* and *Pro*_*35S*_*::OsMADS51-mCherry* were co-transformed to rice protoplasts by polyethylene glycol-mediated transformation (Bart *et al.*, 2006). Fluorescence signals were observed by confocal laser scanning microscopy (LSM 700; Carl Zeiss). Root tissues of *Pro*_*OsHAPL1*_*:: Genome*_*OsHAPL1*_*-GFP* transgenic plants were also analysed using the confocal laser scanning microscope with *Pro*_*35S*_*::GFP* transgenic plants as controls.

### Yeast two-hybrid assays

Yeast two-hybrid assays were carried out to detect the interaction of relevant proteins using a procedure provided by Clontech. Baits consisted of proteins fused with the GAL4 DNA binding domain in pGBKT7 (Clontech). Preys were constructed by fusing proteins with the GAL4 activation domain from pGADT7. Bait and prey were co-transformed into yeast (*Saccharomyces cerevisiae*) AH109 cells. Positive interactions were confirmed by the ability of AH109 cells to grow on Synthetic Dropout/-Trp-Leu-His-Ade medium in a 30°C incubator.

### *In vitro* pull-down assays

Plasmids containing GST-, HIS-, and MBP-labelled proteins were transformed into *Escherichia coli* strain BL21. Transformants were grown to a concentration of OD_600_ = 0.6 in a 37°C concentrator, and placed in a 22°C concentrator for at least 30 min. Expression of the fusion protein was then induced by adding isopropyl β-D-1-thiogalactopyranoside to 0.5 mM, and shaking for 5–6 h in the 22°C table concentrator. *E. coli* cells were lysed in Tris-HCl buffer containing 25 mM Tris·HCl (pH 7.5), 150 mM NaCl, and 1 mM DTT. The sample liquid was then sonicated to destroy the cell (cycles of 3 s on, 3 s off for a total time of 2 min). If the resulting bacterial liquid was not clear, sonication was repeated. Equal amounts of the two protein solutions were mixed and centrifuged, and the supernatant was incubated with glutathione agarose beads (GE Healthcare) overnight at 4°C. After spinning (500 *g*, 5 min, 4°C), the beads were collected and washed five times with buffer containing 25 mM Tris·HCl (pH 7.5), 150 mM NaCl, 1 mM DTT, 1% Triton X100, and 0.1% SDS. Finally, the proteins bound to the beads were separated from the beads by boiling in 2×SDS loading buffer in a 95–100°C water bath. The proteins were separated in SDS-PAGE gels and detected by western blot analysis using anti-GST (Medical Biological Laboratories,1:2000) and anti-MBP antibodies (New England Biolabs, 1:2000).

### Bimolecular fluorescence complementation assays

The interacting proteins (OsHAPL1 and DTH8, DTH8 and Hd1) referred to in this paper were separately fused with YFPC and YFPN from the expression plasmids pSPYCE(M) and pSPYNE173. The plasmids were introduced into *Agrobacterium tumefaciens* (strain GV3101) and infiltrated into tobacco (*Nicotiana benthamian*a) according to the protocol described by Walter *et al.* (2004). Injected leaves were visualized by laser scanning microscopy (LSM 700; Carl Zeiss) 3 days after infiltration.

### Yeast one-hybrid assay

The coding region of *OsHAPL1* was inserted into the pB42AD vector. The promoter region of *Ehd1* were amplified and inserted into the pLacZi vector. These constructs were transformed into the yeast strain EGY48. The assay was performed as previously described (Gao *et al.*, 2013).

### Chromatin immunoprecipitation assay

ChIP assay was performed as previously described (Zhang *et al.*, 2012). Anti-DDDDK-tag antibodies (Medical Biological Laboratories, PM020) were used for detection.

## Results

### Constitutive expression of the *OsHAPL1* gene causes delayed flowering

**Fig. 1.**
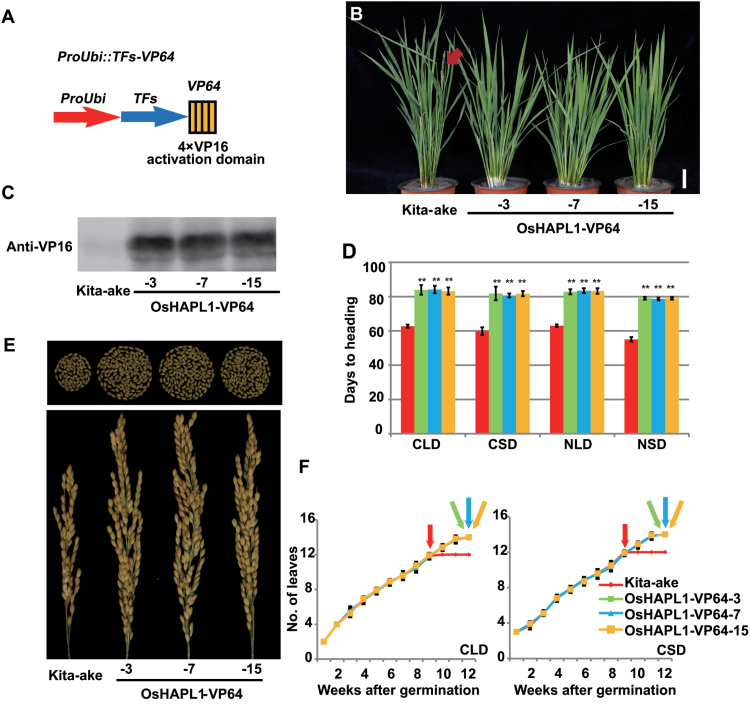
**Phenotypic characterization of OsHAPL1-VP64 transgenic plants.** (**A**) Diagram of *Pro*_*Ubi*_*::TFs-VP64*. (**B**) Heading date phenotypes of OsHAPL1-VP64 transgenic plants under LD conditions. Red arrow indicates heading. (**C**) Protein expression levels of OsHAPL1 in Kita-ake and three independent OsHAPL1-VP64 transgenic plants. (**D**) Statistical data for days to heading of Kita-ake and OsHAPL1-VP64 transgenic plants under different light conditions. See (F) for colour codes. (Student’s *t*-test: **P < 0.01) (**E**) Grains from a single panicle sampled from fully matured plants and panicle morphology of Kita-ake and OsHAPL1-VP64 transgenic lines. (**F**) Rate of leaf emergence of Kita-ake and transgenic plants under CLD and CSD conditions (*n* = 20). Arrows indicate approximate flowering time points of various lines. CLD, controlled long days; CSD, controlled short days; NLD, natural long days; NSD, natural short days.

Previous work has investigated the regulatory mechanism of photoperiodically determined flowering date by large-scale screening of transcriptional factors (TFs) related to flowering (Zhao *et al.*, 2015; Tan *et al.*, 2016). We fused different types of TFs to the VP64 activation domain, driven by the maize (*UBI*) promoter (Fig. 1A). These constructs were then transformed into *japonica* rice cultivar cv. Kita-ake. This cultivar can be transformed at high efficiency and has a very short life span due to defects in several heading date repressors; this facilitates rapid screening of flowering control genes (Xue *et al.*, 2008; Gao *et al.*, 2013; Koo *et al.*, 2013; Kwon *et al.*, 2015). In addition, using Kita-ake can help to identify regulatory networks independent of the repressors known to be defective in Kita-ake. Previous studies have shown that TFs-VP64 in this system can cause strong dominant effects, allowing the function of the TFs to be studied (Beerli *et al.*, 1998; Hanano and Goto, 2011). More than 50 TFs affecting flowering time have previously been isolated (Sheng *et al.*, 2016; Tan *et al.*, 2016). From these 50 candidate TFs, we focused on a new gene, *LOC_Os05g41450*, with unknown function. Conserved domains analysis at the National Center for Biotechnology Information showed there was a conserved H2A superfamily domain, which is same as the transcription factor CBF/NF-Y/archaeal histone domain analysed at http://www.ebi.ac.uk/interpro/ (see Supplementary Fig. S1A, available at *JXB* online). Phylogenetic tree analysis showed that this protein is homologous to HAP2 subunit members (Supplementary Fig. S1B), so we named it OsHAPL1 (HAP2-LIKE 1).

In order to confirm the function of OsHAPL1 in flowering, we studied the phenotypes of OsHAPL1-VP64 transgenic plants. We randomly selected three independent homozygous transgenic lines to check the protein expression level; there was clear overexpression of OsHAPL1 (Fig. 1C). Transgenic plants were late heading compared to the Kita-ake wild type (WT) grown under NLD conditions (Fig. 1B). To determine whether the heading date of OsHAPL1-VP64 transgenic plants was affected by photoperiod, the plants were grown under CLD, CSD, NLD, and NSD conditions. The heading date of the transgenic plants was about 20 days later than that of Kita-ake plants under all four test conditions (Fig. 1D). OsHAPL1-VP64 transgenic plants had larger panicles and more grains than WT (Fig. 1E). Other agronomic traits, such as plant height, tiller number, panicle length, primary branches per panicle, secondary branches per panicle, grains per panicle, and 1000-grain weight were significantly increased relative to WT (Table 1). Leaf emergence rates were checked to eliminate the effect of vegetative stage on the heading date. There was no difference between these lines (Fig. 1F).

**Table 1.** Phenotypic data for Kita-ake, and OsHAPL1-VP64 transgenic plants

Genotype	Condition	Days to flowering	Plant height, cm	Tillers	Panicle length, cm	Primary branches n per panicle	Secondary branches n per panicle	Grains per panicle	1000-grain weight, g
Kita-ake	NLD	63.05 ± 0.76	70.95 ± 1.96	19.25 ± 2.79	14.23 ± 0.6	7.10 ± 0.72	11.50 ± 1.76	70.75 ± 6.38	27.07 ± 0.35
OsHAPL1-VP64-3	NLD	82.80 ± 1.51	93.15 ± 2.70	11.05 ± 1.67	17.42 ± 0.82	14.45 ± 1.32	21.50 ± 4.03	143.85 ± 7.81	27.09 ± 0.57
OsHAPL1-VP64-7	NLD	83.45 ± 1.39	92.70 ± 2.00	10.20 ± 0.95	17.02 ± 0.78	14.05 ± 1.36	21.25 ± 2.88	143.60 ± 6.64	26.44 ± 0.66
OsHAPL1-VP64-15	NLD	83.22 ± 1.64	92.80 ± 2.58	10.10 ± 0.84	17.25 ± 0.95	15.02 ± 1.96	22.74 ± 4.26	140.20 ± 9.36	27.53 ± 1.07
Kita-ake	NSD	55.05 ± 1.31	59.32 ± 2.65	19.95 ± 1.93	10.58 ± 0.80	5.37 ± 0.60	5.21 ± 1.65	41.53 ± 5.85	26.31 ± 0.59
OsHAPL1-VP64-3	NSD	79.07 ± 0.80	97.60 ± 1.84	11.53 ± 1.64	16.86 ± 0.90	13.9 ± 0.99	19.10 ± 1.73	132.40 ± 4.67	26.80 ± 0.54
OsHAPL1-VP64-7	NSD	78.67 ± 0.72	99.40 ± 2.61	11.93 ± 1.10	17.98 ± 0.60	15.1.±1.52	27.50 ± 5.04	162.80 ± 10.57	27.08 ± 0.67
OsHAPL1-VP64-15	NSD	79.00 ± 0.68	98.07 ± 2.59	11.43 ± 1.50	17.51 ± 1.06	14.71 ± 1.49	24.07 ± 6.15	149.07 ± 17.88	26.90 ± 0.65

Agronomic traits are based on Kita-ake and three independent homozygous OsHAPL1-VP64 transgenic plants under natural short-day (NSD) and natural long-day (NLD) conditions. Data for days to heading and other agronomic traits are presented as mean ± standard deviation (*n* = 22).

**Fig. 2.**
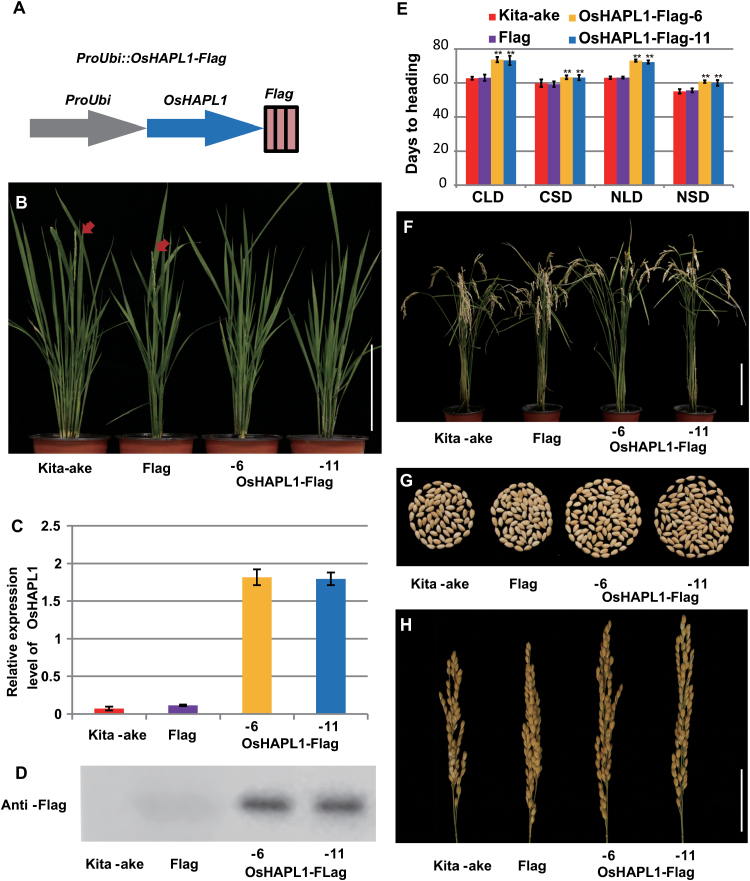
**Phenotypic characterization of OsHAPL1-Flag transgenic plants.** (**A**) Diagram of *Pro*_*Ubi*_*::OsHAPL1-Flag*_._ (**B**) Heading date phenotypes of OsHAPL1-Flag transgenic plants under LD conditions. Red arrows indicate heading. (**C, D**) mRNA (C) and protein (D) expression levels of *OsHAPL1* in Kita-ake, Flag, and two independent OsHAPL1-Flag transgenic plants. (**E**) Statistical data for wild-type Kita-ake, Flag, and OsHAPL1-Flag transgenic plants under different light conditions. (Student’s *t*-test: **P < 0.01). (**F**) Mature stage of Kita-ake, Flag, and OsHAPL1-Flag transgenic plants under NLD conditions. (**G**) Grains from a single panicle sampled the fully matured plants of Kita-ake, Flag, and OsHAPL1-Flag transgenic plants. (**H**) Panicle morphology of Kita-ake and OsHAPL1-Flag transgenic plants. CLD, controlled long days; CSD, controlled short days; NLD, natural long days; NSD, natural short days.

To further confirm the function of OsHAPL1 for flowering, the constitutive expression vector Pro_Ubi_::OsHAPL1-Flag was constructed and transformed into Kita-ake WT (Fig. 2A). We randomly selected two independent homozygous transgenic lines to determine the mRNA and protein expression levels; there was obvious mRNA and protein overexpression of OsHAPL1 (Fig. 2C, D). The transgenic plants were late flowering relative to Kita-ake and Pro_ubi_::Flag (control) transgenic plants under NLD conditions (Fig. 2B). Similarly, to check whether the heading dates of OsHAPL1-Flag transgenic plants were affected by photoperiod, heading date was obtained under different conditions. The transgenic plants had later heading dates than Kita-ake and Pro_ubi_::Flag transgenic plants in all four environments (Fig. 2E). Transgenic plants were taller and produced larger panicles with more grains (Fig. 2F, G, H). Other agronomic traits were similar to those of OsHAPL1-VP64 transgenic plants (Table 2). These data show that OsHAPL1 functions as a flowering repressor.

**Table 2.** Phenotypic data for Kita-ake, Flag, and OsHAPL1-Flag transgenic plants

Genotype	Condition	Days to flowering	Plant height, cm	Tillers	Panicle length, cm	Primary branches n per panicle	Secondary branches per panicle	Grains per panicle	1000-grain weight, g
Kita-ake	NLD	63.05 ± 0.76	70.95 ± 1.96	19.25 ± 2.79	14.23 ± 0.6	7.10 ± 0.72	11.50 ± 1.76	70.75 ± 6.38	27.07 ± 0.35
Flag	NLD	63.17 ± 0.58	71.08 ± 2.15	18.83 ± 3.16	14.25 ± 0.64	7.08 ± 0.67	11.64 ± 1.44	69.33 ± 5.26	26.94 ± 0.36
OsHAPL1-Flag-6	NLD	73.15 ± 0.67	76.10 ± 3.40	14.65 ± 2.78	16.07 ± 0.42	10.15 ± 1.6	15.70 ± 3.47	99.20 ± 11.06	26.94 ± 0.72
OsHAPL1-Flag-11	NLD	72.30 ± 0.98	74.20 ± 2.98	15.42 ± 2.99	15.94 ± 0.46	11.52 ± 2.25	16.35 ± 4.66	95.50 ± 8.53	27.04 ± 1.43
Kita-ake	NSD	55.05 ± 1.31	59.32 ± 2.65	19.95 ± 1.93	10.58 ± 0.80	5.37 ± 0.60	5.21 ± 1.65	41.53 ± 5.85	26.31 ± 0.59
Flag	NSD	55.64 ± 1.21	59.27 ± 2.37	20.27 ± 1.85	10.59 ± 0.97	5.18 ± 0.40	5.09 ± 1.58	41.36 ± 5.43	26.09 ± 0.86
OsHAPL1-Flag-6	NSD	61.40 ± 0.70	77.10 ± 2.42	12.70 ± 2.00	12.84 ± 0.81	6.63 ± 0.92	9.50 ± 1.60	59.50 ± 8.52	26.31 ± 0.59
OsHAPL1-Flag-11	NSD	60.01 ± 0.88	74.50 ± 3.44	12.20 ± 4.10	12.25 ± 0.65	6.13 ± 0.64	8.75 ± 1.91	56.00 ± 8.00	26.81 ± 0.56

Agronomic traits are based on Kita-ake, Flag, and two independent homozygous OsHAPL1-Flag transgenic plants under natural short-day (NSD) and natural long-day (NLD) conditions. Data for days to heading and other agronomic traits are presented as mean ± standard deviation (*n* = 25).

### Downregulation of the *OsHAPL1* gene causes early flowering in long-day conditions

**Fig. 3.**
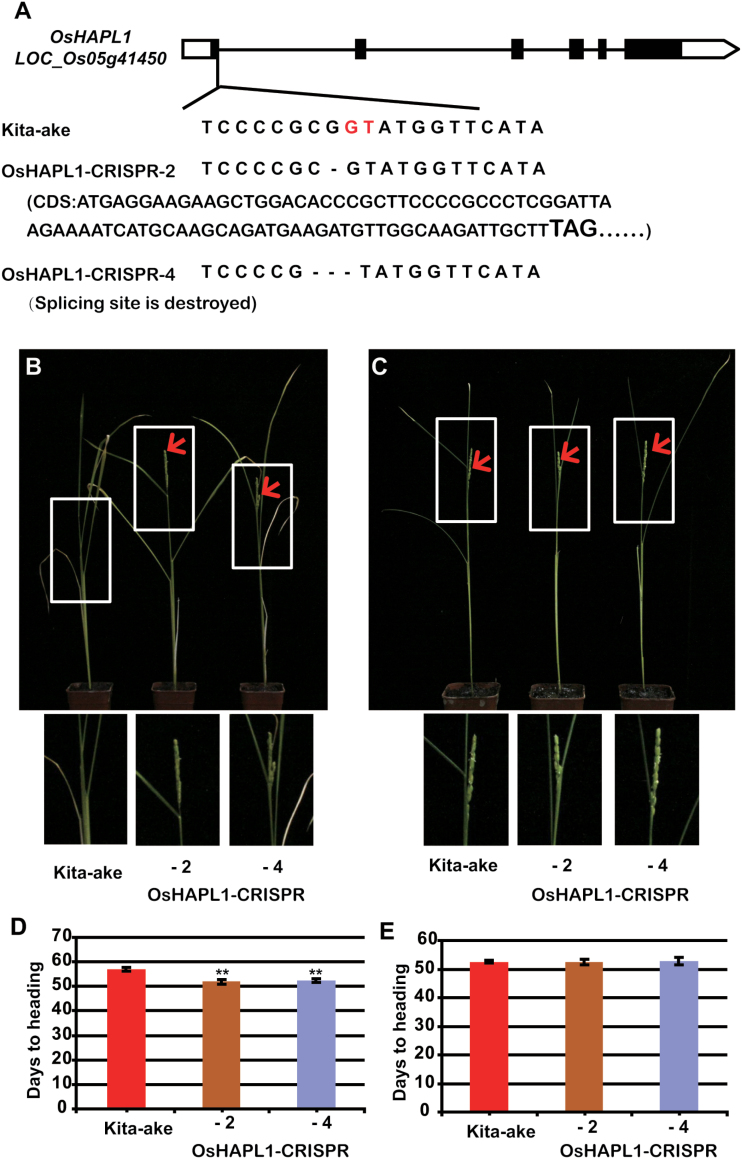
**The phenotype of *oshapl1* mutants.** (**A**) Mutant site in *oshapl1* induced by the CRISPR-*Cas9* genome editing system. The one-base deletion in line 2 resulted in an early stop codon (bold letters), and the three-base deletion in line 4 disrupted the slicing site. The splicing site is highlighted in red. (**B**) Phenotype of *oshapl1* mutants under CLD conditions. A close-up of the top part of the corresponding plants is shown below the panel. Red arrows indicate heading. (**C**) Phenotype of *oshapl1* mutants under CSD conditions. A close-up of the top part of the corresponding plants is shown below the panel. Red arrows indicate heading. (**D**) Statistical data for heading date of Kita-ake and *oshapl1* mutants under CLD. (**E**) Statistical data for heading date of Kita-ake and *oshapl1* mutants under CSD. CLD, controlled long days; CSD, controlled short days; NLD, natural long days; NSD, natural short days.

To further confirm the function of OsHAPL1, an *oshapl1* mutant was constructed using the CRISPR-*Cas9* method (Miao *et al.*, 2013). Two homozygous *oshapl1* mutant lines were obtained (Fig. 3A). These plants flowered earlier than WT under CLD conditions (Fig. 3B, D) but not under CSD conditions (Fig. 3C, E). We also created *oshapl1* mutants using the same method in Nipponbare, a day length-sensitive cultivar, and observed a similar earlier flowering phenotype under NLD conditions (see Supplementary Fig. S2, available at *JXB* online). Thus, OsHAPL1 has a role in controlling flowering time under LD.

### OsHAPLl is a nuclear-localized protein and highly expressed in leaves

**Fig. 4.**
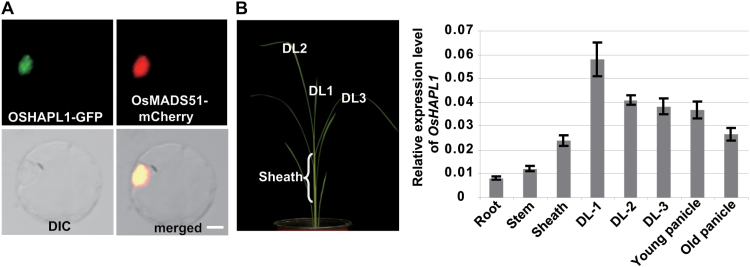
**Properties of OsHAPL1 protein and expression pattern of *OsHAPL1*.** (**A**) Subcellular localization of OsHAPL1 in the rice protoplast. Scale bar = 5 μm. (**B**) Expression pattern of *OsHAPL1* in different tissues. DL1 (uppermost leaf), DL2 (second uppermost leaf), DL3 (third uppermost leaf), and leaf sheath were sampled as shown in the left picture.

To investigate the subcellular localization of OsHAPLl, the OsHAPLl-GFP fusion protein was introduced into rice protoplasts. GFP fluorescence signals were detected in the nucleus, and the signals merged with known nuclear-localized signals (Fig. 4A). GFP signals were also detected in the nucleus of root cells of *Pro*_*OsHAPL1*_*::Genomic*_*OsHAPL1*_*-GFP* transgenic rice (see Supplementary Fig. S3, available at *JXB* online).

Real-time qRT-PCR assays were performed with mRNA from various tissues to analyse the expression patterns of *OsHAPL1*. *OsHAPL1* was generally expressed in all tissues, but more highly expressed in the uppermost and second uppermost leaves (DL1, DL2) (Fig. 4B). High expression of *OsHAPL1* in leaves is consistent with functions related to heading date.

### Expression of *OsHAPL1* is photoperiod responsive

**Fig. 5.**
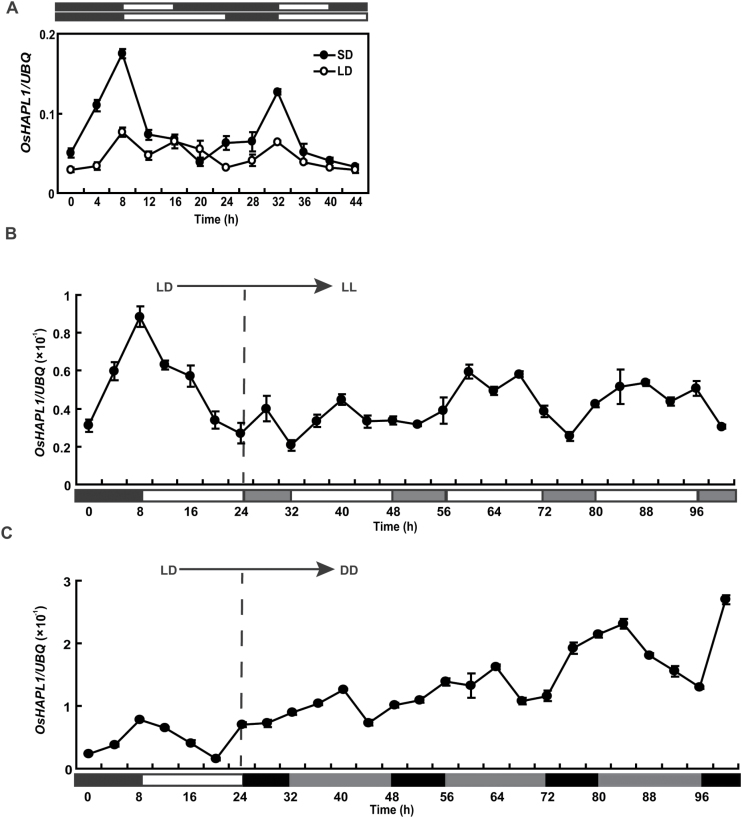
**Expression analysis of *OsHAPL1*.** (**A**) Rhythmic expression of *OsHAPL1*. (**B, C**) Expression analysis of *OsHAPL1* under continuous light (LL) (B) and continuous dark (DD) (C) conditions. The plants were grown in growth chambers under LD conditions (14 h light /10 h darkness) for 30 days and then transferred to LL or DD conditions. White bars indicate light; black bars indicate darkness. The rice *Ubiquitin* gene was used as the internal control. Values represent means ± standard deviation (SD) from three independent biological replicates.

*OsHAPL1* exhibited the same diurnal rhythmic expression patterns under SD and LD conditions. It accumulated from the beginning of darkness, reached a peak at the start of the light period, and then rapidly decreased (Fig. 5A). To ascertain if *OsHAPL1* transcript levels were controlled by the circadian clock, plants grown in LD conditions were transferred to continuous light or continuous darkness, and then checked for expression of *OsHAPL1*. When subjected to continuous darkness or light conditions, the rhythmic expression patterns disappeared (Fig. 5B, C). Weakening of the rhythm in continuous darkness and continuous light suggests that *OsHAPL1* is not controlled by the circadian clock. These results indicate instead that *OsHAPL1* is expressed rhythmically and affected by photoperiod.

### OsHAPL1 interacts with DTH8-Ghd8-OsHAP3H

To investigate the potential functional role of OsHAPLl on heading date, we looked for proteins that interact with OsHAPLl using yeast two-hybrid screening of the yeast strain AH109. Using pGBKT7-OsHAPLl (a fusion protein consisting of the GAL4 DNA binding domain and OsHAPLl) as bait, we identified an amino acid sequence corresponding to residues 3–125 of DTH8-Ghd8-OsHAP3H (LOC-Os08g07740.1) (Wei *et al.*, 2010; Yan *et al.*, 2011) from a rice GAL4 activation fusion library. Previous studies have shown that DTH8 is a CCAAT-box-binding TF encoding a putative HAP3 subunit, and has a role in determining heading date in rice. Overexpression of DTH8 delays flowering (Wei *et al.*, 2010); this phenotype is consistent with overexpression of OsHAPL1.

**Fig. 6.**
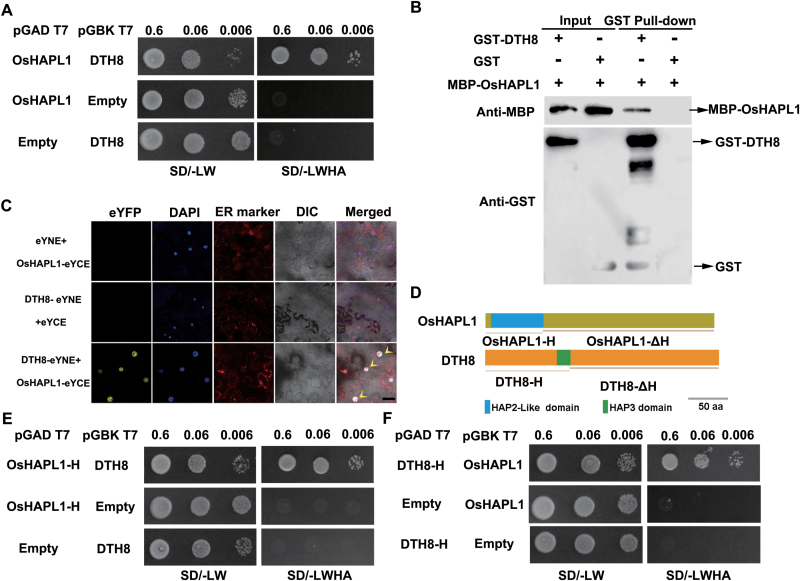
**Interactions between OsHAPL1 and DTH8.** (**A**) OsHAPL1 interacted with DTH8 in yeast two-hybrid assays. Yeast was grown at 30°C for 4 days. (**B**) OsHAPL1 interacted with DTH8 in GST pull-down assays. Arrow indicates the target protein. (**C**) OsHAPL1 interacted with DTH8 in tobacco BiFC assays. Scale bar = 20 μm. (**D**) Protein structural diagram of OsHAPL1 and DTH8. aa, amino acids. (**E**) OsHAPL1 interacted with DTH8 in yeast two-hybrid assays owing to its HAP2-like domain. Yeast was grown at 30°C for 4 days. (**F**) DTH8 interacted with OsHAPL1 in yeast two-hybrid assays owing to its HAP3 domain. Yeast was grown at 30°C for 4 days. DAPI, 4'-6-diamidino-2-phenylindole; DIC, differential interference contrast microscope; ER, endoplasmic reticulum; eYCE, pSPYCE(M); eYFP, enhanced yellow fluorescent protein; eYNE, pSPYNE173; SD/-LW, Synthetic Dropout/-Trp-Leu; SD/-LWHA, Synthetic Dropout/-Trp-Leu-His-Ade.

To confirm the results from library screening, the interaction between the full-length amino acid sequences of OsHAPLl and DTH8 was tested on selective media plates (Fig. 6A). An *in vitro* pull-down assay was performed to further confirm the interaction of OsHAPLl and DTH8. GST-tagged DTH8 could pull down MBP-tagged OsHAPLl, whereas GST alone could not pull down MBP-tagged OsHAPLl (Fig. 6B). To investigate whether OsHAPLl interacts with DTH8 *in planta*, a BiFC assay was performed in *N. benthamiana*. Fluorescence signals were observed in the epidermal cells of tobacco leaves transformed transiently with pSPYCE(M)-OsHAPLl and pSPYNE173-DTH8 (Fig. 6C), whereas no signals were present in the negative control (transformed with pSPYCE(M)-OsHAPLl and pSPYNE173, pSPYCE(M) and pSPYNE(173)-DTH8) (Fig. 6C). We also used qRT-PCR to investigate the expression of *DTH8* and found that *DTH8* has a similar diurnal expression pattern to *OsHAPL1* (see Supplementary Fig. S4, available at *JXB* online). Together, these results suggest a possible interaction *in planta* between OsHAPLl and DTH8.

To identify the OsHAPLl domain involved in the interaction with DTH8, we performed yeast two-hybrid assays using either OsHAPLl with the HAP domain only (OsHAPLl-H) or OsHAPLl with the HAP domain deleted (OsHAPLl-∆H) and DTH8 (Fig. 6D). OsHAPLl-H interacted with the DTH8 protein, whereas OsHAPLl-∆H did not (Fig. 6E). DTH8 also interacted with OsHAPLl by means of its HAP domain (Fig. 6F). These experiments indicate that the HAP domain is essential for the interaction between OsHAPLl and DTH8.

### OsHAPL1, Hd1, and DTH8 form a complex in *E. coli*

**Fig. 7.**
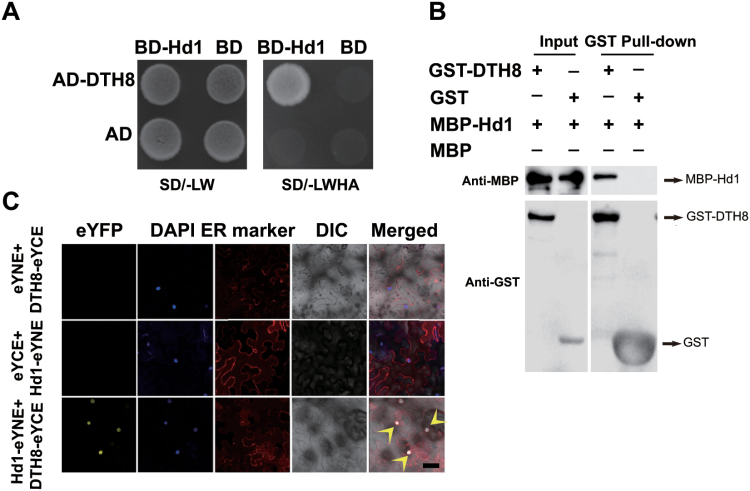
**Interaction between DTH8 and Hd1.** (**A**) DTH8 interacted with Hd1 in yeast two-hybrid assays. Yeast was grown at 30°C for 4 days. (**B**) DTH8 interacted with Hd1 in GST pull-down assays. Arrows indicate target proteins. (C) OsHAPL1 interacted with DTH8 in tobacco BiFC assays. Scale bar = 20 μm. DAPI, 4'-6-diamidino-2-phenylindole; DIC, differential interference contrast microscope; ER, endoplasmic reticulum; eYCE, pSPYCE(M); eYFP, enhanced yellow fluorescent protein; eYNE, pSPYNE173; SD/-LW, Synthetic Dropout/-Trp-Leu; SD/-LWHA, Synthetic Dropout/-Trp-Leu-His-Ade.

Hd1 is an important protein involved in determining the heading date in rice (Yano *et al.*, 2000). Hd1 can delay flowering in LD conditions, and this function is consistent with those of OsHAPL1 and DTH8, so we speculated whether Hd1 can interact with OsHAPL1 and DTH8. A yeast two-hybrid assay showed that Hd1 cannot interact with OsHAPL1 (data not shown), where it can interact with DTH8 (Fig. 7A). We further confirmed the interaction between Hd1 and DTH8 by *in vitro* pull-down and BiFC assays (Fig. 7B, C).

**Fig. 8.**
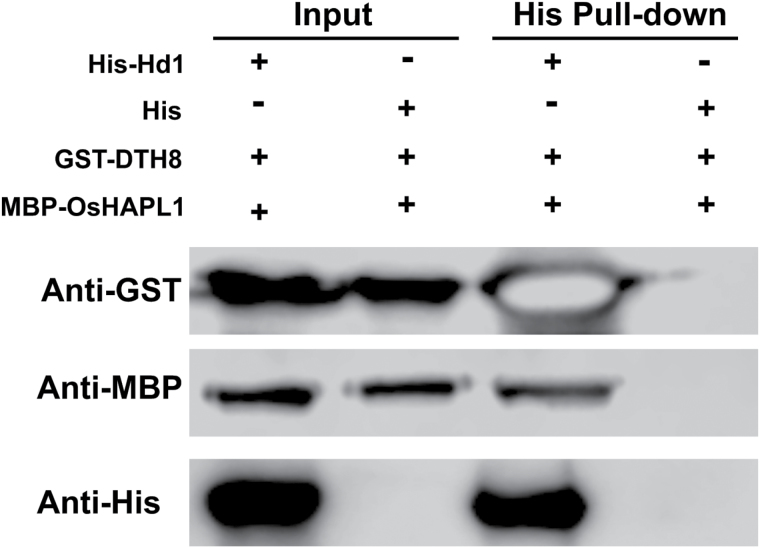
**OsHAPL1, DTH8, and Hd1 form a complex in His pull-down assays.** *In vitro* binding assay with the indicated recombinant proteins showed direct interaction between OsHAPL1, DTH8, and Hd1.

The interaction among OsHAPL1, DTH8, and Hd1 implies that these three proteins may form a complex, and that DTH8 may function as a bridge. To confirm this, histidine pull-down of these three proteins was performed. DTH8 and OsHAPL1 were pulled down by Hd1 (Fig. 8), suggesting that OsHAPL1, DTH8, and Hd1 can form a complex in *E. coli*.

### OsHAPL1, DTH8, and Hd1 interact with HAP family proteins in yeast

**Fig. 9.**
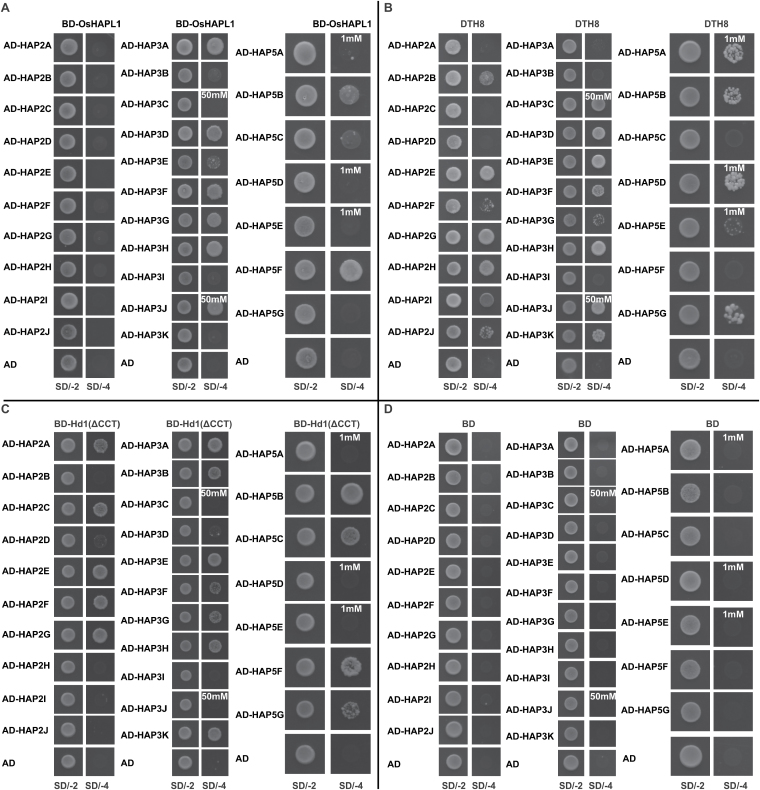
**Interactions between OsHAPL1, DTH8, Hd1, and HAP subunit members.** (**A**) Interactions between OsHAPL1 and HAP in yeast two-hybrid assays. (**B**) Interactions between DTH8 and HAP in yeast two-hybrid assays. (**C**) Interactions between Hd1 and HAP in yeast-two-hybrid assays. (**D**) Negative control. AD, pGAD T7; BD, pGBK T7; SD/-2, Synthetic Dropout/-Trp-Leu; SD/-4, Synthetic Dropout/-Trp-Leu-His-Ade.

Previous studies have shown that various HAP complexes control different growth and developmental processes by tissue-specific expression and complex formation of the three subunits (Thirumurugan *et al.*, 2008; Laloum *et al.*, 2013). To preliminarily identify the possible HAP members involved in the heading date related to OsHAPL1, DTH8, and Hd1, we performed yeast two-hybrid assays. OsHAPL1 interacted with HAP3 family members HAP3A, HAP3D, HAP3F, HAP3G, HAP3H, and HAP3J, and with HAP5 members HAP5B and HAP5F (Fig. 9A). DTH8 interacted with HAP2 family members HAP2B, HAP2E, HAP2G, HAP2H, HAP2I, and HAP2J; with HAP3 members HAP3C, HAP3D, HAP3E, HAP3F, HAP3H, HAP3J, and HAP3K; and with HAP5 members HAP5A, HAP5B, HAP5D, and HAP5G (Fig. 9B). Because Hd1 can self-activatie, Hd1(ΔCCT) fused with the GAL4 DNA binding domain. Yeast data showed that Hd1(ΔCCT) can interact with HAP2 family members HAP2A, HAP2C, HAP2E, HAP2F, and HAP2G; with HAP3 members HAP3A, HAP3B, HAP3E, HAP3F, HAP3G, HAP3H, and HAP3K; and with HAP5 members HAP5B, HAP5C, HAP5F, and HAP5G (Fig. 9C). Figure 9D shows the negative control. These data suggest many HAP family members can interact with OsHAPL1, DTH8, and Hd1, and suggest that these HAP members may be involved in the heading date.

### OsHAPL1, DTH8, and Hd1 interact with components of the transcription start complex in yeast

**Fig. 10.**
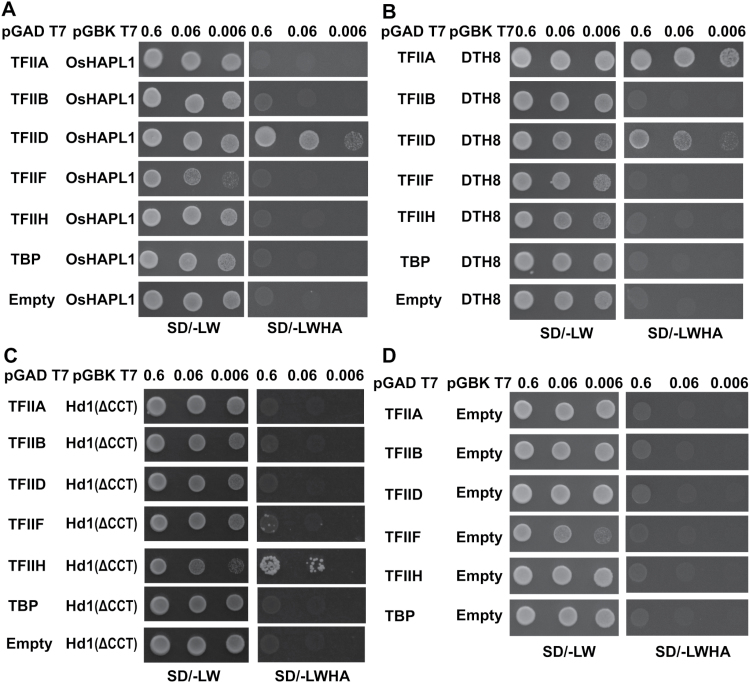
**Interactions between OsHAPL1, DTH8, Hd1, and GTFs.** (**A**) Interactions between OsHAPL1 and GTFs in yeast two-hybrid assays. (**B**) Interactions between DTH8 and GTFs in yeast two-hybrid assays. (**C**) Interactions between Hd1 and GTFs in yeast two-hybrid assays. (**D**) Negative control. SD/-LW, Synthetic Dropout/-Trp-Leu; SD/-LWHA, Synthetic Dropout/-Trp-Leu-His-Ade.

Because a previous study indicated that the interaction of HAP3 and HAP5 with TATA-Binding Protein (TBP) depends on short domains adjacent to their histone fold motifs (Bellorini *et al.*, 1997), we speculated that HAP proteins could be involved in transcriptional start complex formation. To verify this possibility, we performed a yeast two-hybrid assay to determine whether OsHAPL1 can interact with GTFs. Results showed that OsHAPL1 could interact with TFIID but not with TFIIA, TFIIB, TFIIF, TFIIH or TBP (Fig. 10A). Because OsHAPL1, DTH8, and Hd1 can form a complex, we speculated that DTH8 and Hd1 could also interact with GTFs. DTH8 interacted with TFIIA and TFIID, but could not interact with TFIIB, TFIIF, TFIIH, or TBP (Fig. 10B). Hd1 interacted with TFIIH, but could not interact with TFIIA, TFIIB, TFIID, TFIIF, or TBP (Fig. 10C). Figure 10D shows the negative control. These preliminary data suggest that OsHAPL1, DTH8, and Hd1 serve as co-regulators of transcription.

### OsHAPL1 delays flowering by repressing *Ehd1* as well as the downstream florigen genes *Hd3a* and *RFT1*

**Fig. 11.**
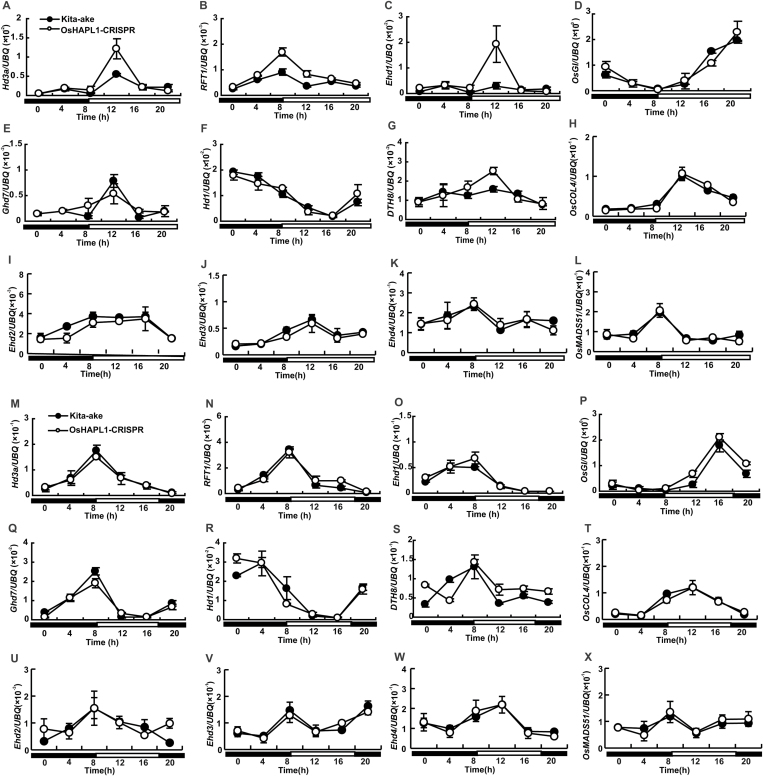
**The rhythmic expression patterns of *Hd3a*, *RFT1*, *Ehd1*, *OsGI*, *Ghd7*, *Hd1*, *DTH8*, *OsCOL4, Ehd2,3,4,* and *OsMADS51* in the wild-type Kita-ake and *oshapl1* mutants under LD conditions (A–L) and SD conditions (M–X).** The plants were grown in growth chambers under LD conditions (14 h light /10 h darkness) and SD conditions (10 h light /14 h darkness) for 30 days. White bars indicate light; black bars indicate darkness. The rice *Ubiquitin* gene was used as the internal control. Values represent means ± standard deviation from three independent biological replicates.

To study downstream genes regulated by OsHAPL1, we analysed the expression of key genes related to the heading date in 30-day-old WT and *oshapl1* mutant plants grown in LD and SD conditions using qRT-PCR. The expression levels of *Ehd1* were upregulated in *oshapl1* mutant plants relative to WT in LD conditions. The florigen genes *Hd3a* and *RFT1* in *oshapl1* mutant plants were also upregulated in LD conditions (Fig. 11). The expression of these three genes was not affected in *oshapl1* mutant plants in SD conditions. The expression of *OsGI*, *Ghd7*, *Hd1*, *DTH8*, *Ehd2/3/4*, *OsMADS51*, and *OsCOL4* was not significantly different between *oshapl1* mutant plants and WT in either environment (Fig. 11). These data suggest that OsHAPL1 can affect heading date via *Ehd1*, *Hd3a*, and *RFT1*. To further confirm that OsHAPL1 functions upstream of *Ehd1*, *Hd3a*, and *RFT1*, we performed expression analysis for *OsHAPL1* in *ehd1*, *hd3a*, and *rft1* mutants. The expression of *OsHAPL1* was not changed in these mutants (see Supplementary Fig. S5, available at *JXB* online). These results suggest that *OsHAPL1* regulates heading date by controlling the expression of *Ehd1*, *Hd3a*, and *RFT1* in LD conditions.

## Discussion

**Fig. 12.**
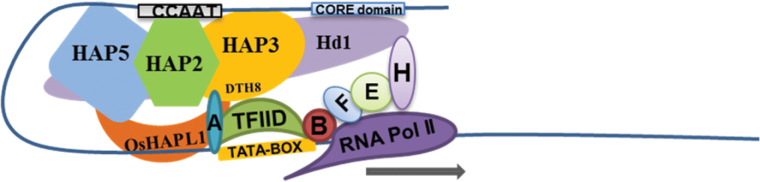
**Proposed model for the OsHAPL1-DTH8-Hd1 complex that affects heading date in rice.** The complex may function as a transcriptional regulator, together with the HAP complex and GTFs, to control expression of downstream genes, and then to affect heading date by influencing the expression of *Hd3a*, *RFT1*, and *Ehd1*.

We report a functional analysis of *OsHAPL1*, which is a novel repressor of heading date. Our studies showed that OsHAPL1 can form a complex with DTH8 and Hd1 in *E. coli*. In addition, OsHAPL1, DTH8, and Hd1 also interact with some members of the HAP subfamily groups and with GTFs. The data strongly indicate that the OsHAPL1-DTH8-Hd1 complex acts as a transcriptional regulator of heading date by interacting with the HAP complex and GTFs (Fig. 12). Furthermore, qPCR analysis of genes related to heading date showed that the expression of *Hd3a*, *RFT1*, and *Ehd1* was downregulated by *OsHAPL1*. Thus, our studies suggest that the OsHAPL1-DTH8-Hd1 complex regulates the transcription of target genes to control heading date in rice.

### OsHAPL1 is a HAP2-like transcription regulator

The HAP complex consists of three subunits, namely HAP2 (also called NF-YA or CBF-B), HAP3 (NF-YB or CBF-A), and HAP5 (NF-YC or CBF-C) (Thirumurugan *et al.*, 2008). Each subunit contains a conserved domain responsible for DNA binding and protein interaction as well as conserved regions (Thirumurugan *et al.*, 2008). The HAP complex binds to CCAAT sequences, which are among the most general trans-acting elements in many genes in fungi, animal, and plants (Maity and de Crombrugghe, 1998; Mantovani, 1999). In yeast, each subunit of HAP2, HAP3, and HAP5 has only one gene-encoded protein, and together they form a heterotrimeric CCAAT-box-binding complex, which can recruit a fourth polypeptide, ScHAP4, that associates with the HAP complex and activates transcription via its acidic domain, but it cannot bind to DNA (Forsburg and Guarente, 1989). In animals, homologues of the yeast HAP complex are called NF-Y, CP1, or CBP (Vuorio *et al.*, 1990; Becker *et al.*, 1991; Li *et al.*, 1992; Sinha *et al.*, 1995); there is no HAP4 homologue and the HAP complex possibly interacts with other TFs to regulate transcription of target genes (Bellorini *et al.*, 1997). In plants each subunit consists of multiple proteins. In rice, there are 11 *HAP2* genes, 12 *HAP3* genes, and 12 *HAP5* genes (Thirumurugan *et al.*, 2008; Li *et al.*, 2016). OsHAPL1, a HAP2-like TF, identified by its effect on heading date, associates with certain members of the HAP3 and HAP5 family proteins. We speculated that OsHAPL1 is a protein homologue of ScHAP4, but blast analysis of the amino acid sequences showed only 9% homology (see Supplementary Fig. S6, available at *JXB* online), indicating they are not homologues. It seems that *OsHAPL1* may function like other HAP family members. In addition, OsHAPL1 also interacts with the GTF TFIID, suggesting that it functions as a transcription regulator in the heading pathway in rice. The CCT domain of CO in Arabidopsis is similar to HAP2 in yeast, and CO may replace AtHAP2 in the HAP complex to form a trimeric CO-AtHAP3-AtHAP5 complex to regulate flowering-related gene expression (Wenkel *et al.*, 2006). When AtHAP3 and AtHAP5 were overexpressed, flowering was delayed. Overexpression of AtHAP2 or AtHAP3 could impair the formation of a CO-AtHAP3-AtHAP5 complex, leading to delayed flowering (Wenkel *et al.*, 2006). Here, OsHAPL1 functioned in the same way as a HAP2-like transcriptional regulator, interacting with some HAP3 and HAP5 proteins to form complexes. DTH8-OsHAP3H and Hd1 can also interact with some HAP2, 3, and 5 proteins. The OsHAPL1-DTH8-Hd1 complex could interact with the HAP complex to regulate the expression of flowering genes, and hence control heading date. More evidence is needed to confirm whether the mechanism is similar to that in Arabidopsis.

### The OsHAPL1-DTH8-Hd1 complex may function as a transcription regulator through direct interaction with general transcription factors

We found that OsHAPL1, DTH8, and Hd1 form a complex in *E. coli*. OsHAPL1 interacts with the GTF TFIID, DTH8 interacts with TFIIA and TFIID, and Hd1 interacts with TFIIH. GTFs and RNA polymerase II play important roles in transcription in eukaryotes. Six GTFs and RNA polymerase II assemble into a stable pre-initiation complex responsible for RNA synthesis (Zawel and Reinberg, 1993). TFIIA plays an important role in transcriptional activation and facilitation of TFIID to its promoter (Buratowski *et al.*, 1989; Maldonado *et al.*, 1990; Ma *et al.*, 1993; Lieberman and Berk, 1994). TFIID binding to the TATA motif helps to bind TFIIB (Buratowski *et al.*, 1989; Maldonado *et al.*, 1990); The TFIID-TFIIB complex then recruits TFIIF and RNA polymerase II to the promoter to form a TFIID-TFIIB-TFIIF-PolII complex intermediate (Flores *et al.*, 1990). TFIIE binds to the promoter by interacting with GTFs, and recruits THIIH to the complex (Flores *et al.*, 1992). The TFIIA-TFIID-TFIIB-TFIIF-PolII-TFIIE-THIIH complex then initiates transcription (Zawel and Reinberg, 1995; Mermelstein *et al.*, 1996). A previous study showed that assembly of the transcription initiation complex requires many regulatory proteins that directly interact with the GTFs (Zawel and Reinberg, 1995). The transcription initiation process also needs many regulatory factors such as activators, repressors, co-activators, and co-repressors (Herschbach and Johnson, 1993). For example, Dr1 in yeast represses transcription of RNA polymerase II and RNA polymerase III (Inostroza *et al.*, 1992; White *et al.*, 1994). Dr1 represses RNA polymerase II transcription because it interacts with TBP and prevents TFIIA and TFIIB entering the pre-initiation complex (Inostroza *et al.*, 1992; Kim *et al.*, 1995), thus preventing formation of an active complex, and initiation of transcription by RNA polymerase II (Inostroza *et al.*, 1992). Dr1-associated protein 1 (DrAp1) forms a heterotetramer with Dr1, but cannot repress transcription. DrAp1 functions as a co-repressor that enhances the repression activity of Dr1 (White *et al.*, 1994; Kim *et al.*, 1995; Mermelstein *et al.*, 1996). dDr1/dDrAp1 in *Drosophila* have a different function, repressing the transcription of TATA-type promoters and activating transcription of a TATA-less promoter (Willy *et al.*, 2000). In rice, Dr1 or DrAp1 separately interact with the TBP-DNA complex, forming heterodimers that strongly interact with the TBP-DNA complex to form larger complexes (Song *et al.*, 2002). OsDrAp1 has strong repression activity and OsDr1 has weak repression activity (Song *et al.*, 2002). OsHAPL1 encodes a histone-like TF, and its homologues in maize and *Brachypodium* are Dr1-associated co-repressors also named OsDrAp2 (Thirumurugan *et al.*, 2008). Taken together, the interaction between OsHAPL1, DTH8, Hd1, and GTFs suggests that OsHAPL1-DTH8-Hd1 may regulate transcription pre-initiation by interacting with some GTFs.

### OsHAPL1-DTH8-Hd1 functions as a repressor of heading in rice under long-day conditions

Heading in rice is regulated by a complicated genetic network involving many genes. Rice is a SD plant that flowers rapidly under SD conditions, but is late flowering under LD conditions (Takimoto and Ikeda, 1961). We found that knockout of *OsHAPL1* promoted flowering under LD conditions, and overexpression of *OsHAPL1* delayed flowering. We also found that OsHAPL1, DTH8, and Hd1 form a complex in *E*. *coli*. Previous studies have shown that Hd1 promotes flowering by regulating the expression of Hd3a (a florigen in rice) in SD conditions, whereas it delays flowering by downregulating expression of Hd3a under LD conditions through unknown mechanisms (Yano *et al.*, 2000; Kojima *et al.*, 2002; Tamaki *et al.*, 2007; Komiya *et al.*, 2008). DTH8 suppresses flowering in rice by downregulating expression of *Ehd1* and *Hd3a* in LD conditions, but has no influence on flowering in SD conditions (Wei *et al.*, 2010). A previous study of *DTH8* also showed a reduced grain number per panicle and yield per plant in the loss-of-function line CSSL61 (Wei *et al.*, 2010). We characterized the yield features by measuring agronomic traits, such as tiller number, grain number per panicle, 1000-grain weight, and yield per plant. We found that grain number per panicle and grain yield per plant were significantly reduced in *oshapl1* (Supplementary Table S2, available at *JXB* online). This is a consistent yield feature between *oshapl1* and *dth8*. Altogether, OsHAPL1, DTH8, and Hd1 function in the same pathway and consistently behave as repressors of heading in LD conditions while having no influence on heading date in SD conditions. The *Ehd1* pathway is a unique, *Hd1*-independent flowering pathway. *Ehd1* encodes a B-type response regulator that is highly conserved in cultivated rice, but has no homologue in Arabidopsis (Doi *et al.*, 2004; Takahashi *et al.*, 2009). It positively regulates expression of *Hd3a* and the florigen *RFT1* (Doi *et al.*, 2004; Komiya *et al.*, 2008; Komiya *et al.*, 2009). Many Ehd1-repressors have already identified, including DTH8. Here, we found another Ehd1-repressor, OsHAPL1, which forms a complex with DTH8 and Hd1, and negatively regulates expression of *Ehd1*, and then inhibits flowering by downregulating expression of *RFT1* and *Hd3a*. Recent studies have shown that Hd1 interacts with Ghd7 to form a complex that can bind to the promoter of *Ehd1* and repress its expression (Nemoto *et al.*, 2016). In our study, Hd1 interacted with DTH8 and OsHAPL1, so we speculate that Hd1, DTH8, OsHAPL1, and Ghd7 may interact with the same complex. Our preliminary yeast data showed that DTH8 can interact with Ghd7, whereas OsHAPL1 cannot (data not shown). Additionally, our ChIP-qPCR and yeast one-hybrid assays showed that OsHAPL1 cannot bind to the promoter of *Ehd1.* OsHAPL1 may regulate other target genes to control heading date via *Ehd1*. Nevertheless, our results have demonstrated the possibility of using *OsHAPL1* to increase the adaptability of rice varieties. Combinational manipulation of this gene, together with other trait genes such as *GS3* for grain size, *Gn1a* for grain number, and *AFD1* for plant height (Ren *et al.*, 2016; Shen *et al.*, 2016), may provide a useful practice for crop improvement.

## Supplementary data

Supplementary data are available at *JXB* online.

Fig. S1. Protein structure and phylogenetic tree of OsHAPL1.

Fig. S2. The phenotype of *oshapl1* mutants in Nipponbare background.

Fig. S3. Subcellular localization of OsHAPL1.

Fig. S4. Rhythmic expression analysis of *DTH8*.

Fig. S5. Expression analysis of *OsHAPL1* between Nipponbare and *ehd1*, *hd3a*, and *rft1* mutants, under CLD and CSD conditions.

Fig. S6. Blast analysis of the amino acid sequences of OsHAPL1 and ScHAP4.

Table S1. Primers used in this study.

Table S2. Comparison of major agricultural traits between Kia-ake and *oshapl1* under NLD conditions.

## Supplementary Material

supplementary_figures_S1_S6_tables_S1_S2
