# Supplementary material for: The OsHAPL1-DTH8-Hd1 complex functions as the transcription regulator to repress heading date in rice
Source: J Exp Bot. 2017 Jan 2;68(3):553–68. doi: 10.1093/jxb/erw468 (PMC6055584; doi:10.1093/jxb/erw468)
Supplement: supplementary_figures_S1_S6_tables_S1_S2 [file erw468_suppl_supplementary_figures_s1_s6_tables_s1_s2.pdf]

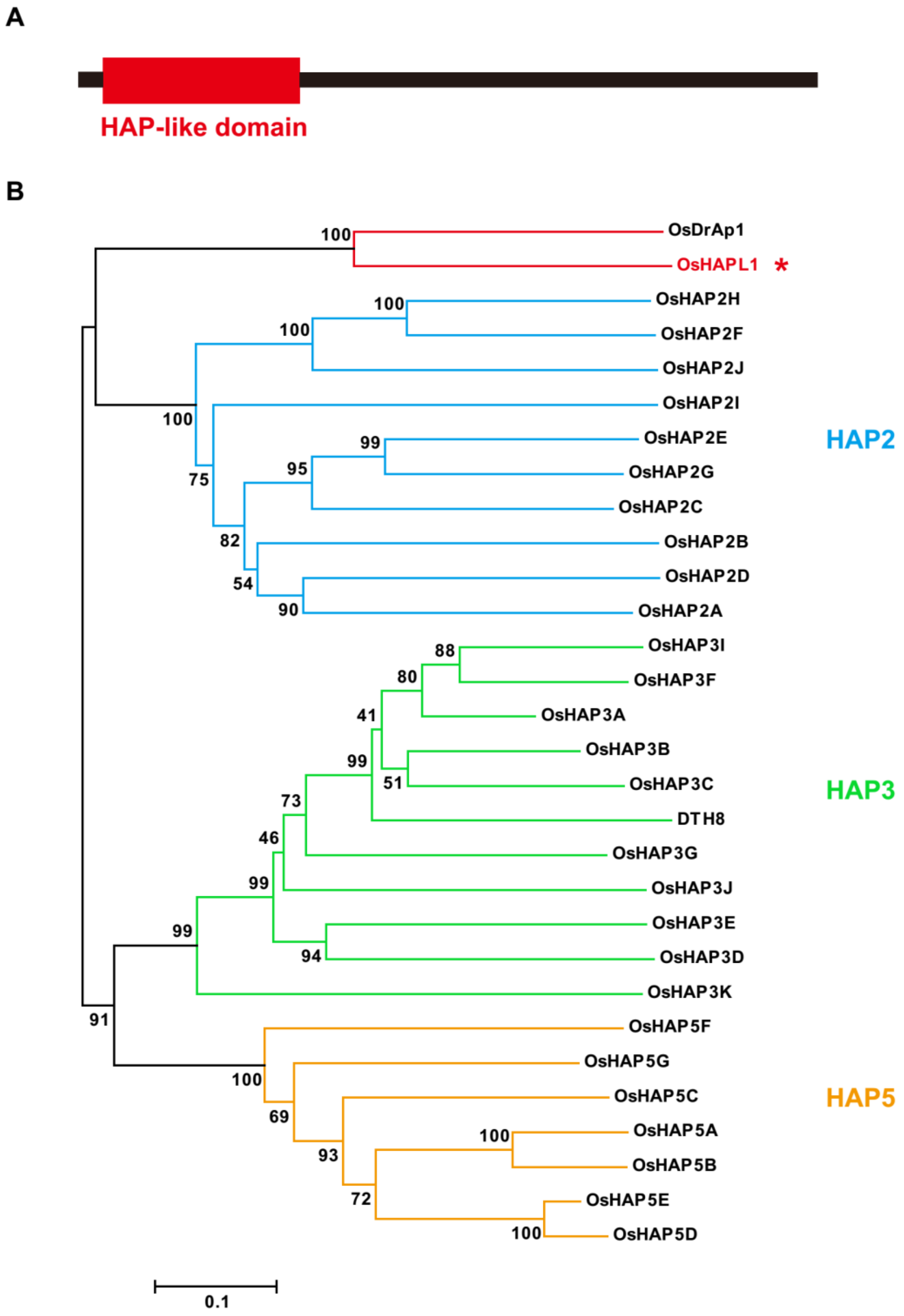

Fig. S1. (A) Protein structure of OsHAPL1.

(B) Phylogenetic tree of the OsHAPL1 and HAP family members in rice. Amino acid sequences of the proteins were obtained from NCBI (http://www.ncbi.nlm.nih.gov/). OsHAPL1 is indicated by an asterisk.

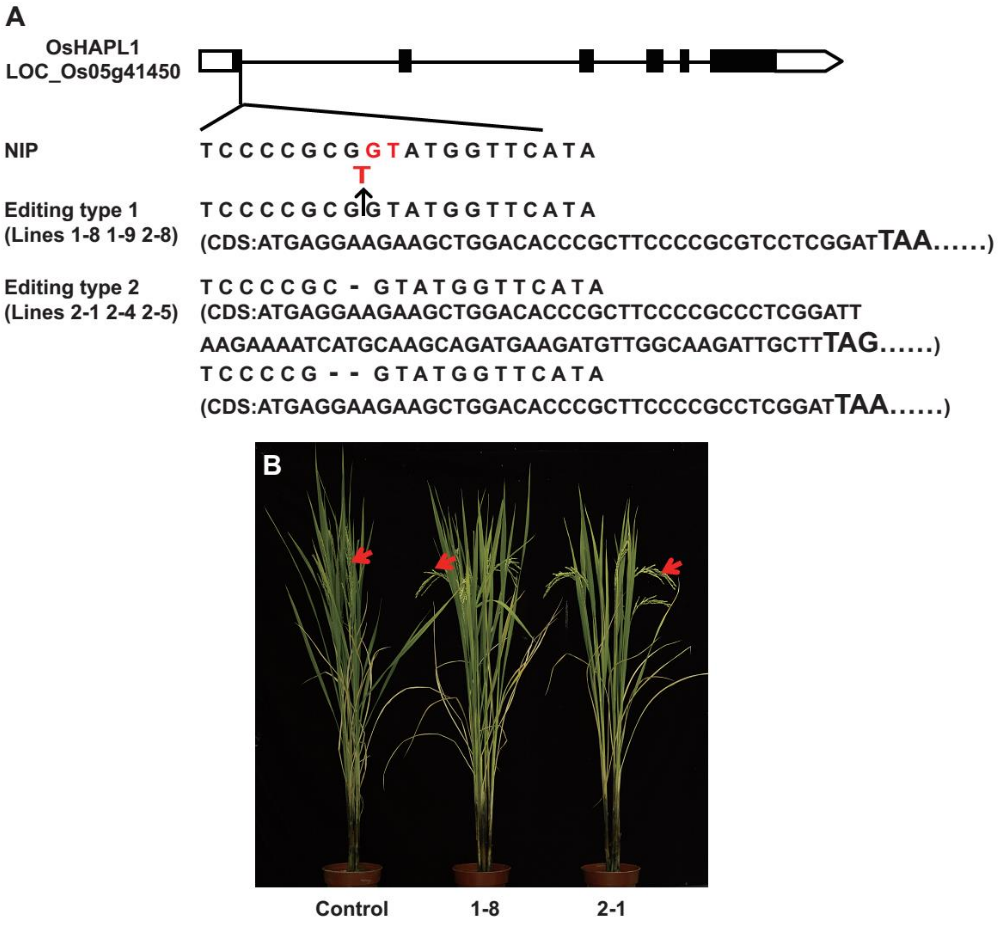

Fig. S2. The phenotype of *oshapl1* mutants in Nipponbare background.

(A) Mutant site in *oshapl1* induced by the CRISPR-*Cas9* genome editing system. The one-base insertion in editing type 1 resulted in an early stop codon (bold letters), and bi-allelic deletions (one base deletion on one chromosome and a two-base deletion on another) in editing type 2 also resulted in an early stop codon (bold letters). The splicing site is highlighted in red. (B) Phenotype of *oshapl1* mutants under NLD conditions.

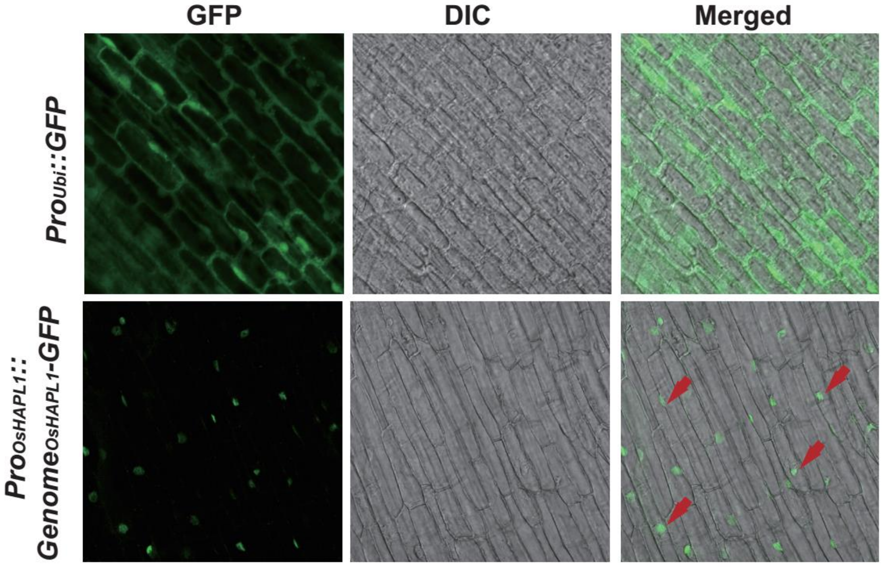

Fig. S3. Subcellular localization of OsHAPL1 in roots of *Pro$_{OsHAPL1}$*:: *Genome$_{OsHAPL1}$-GFP* transgenic rice plants. Red arrows showed nuclear signals.

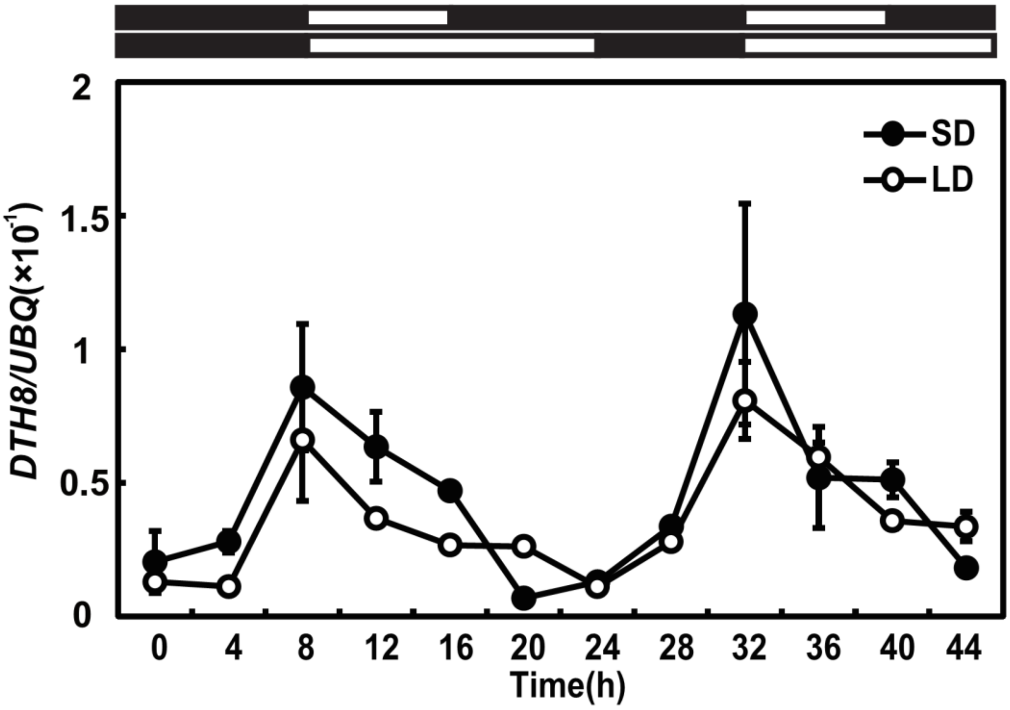

Fig. S4. Rhythmic expression analysis of *DTH8*.

The plants were grown in growth chambers under LDs (14 h light /10 h darkness) and SDs (10 h light /14 h darkness) for 30 days. White bars indicate light; Black bars indicate darkness. The rice *Ubiquitin* (*UBQ*) gene was used as the internal control. Values represent means ± standard deviation from three independent biological replicates. X-axis: Time (h).

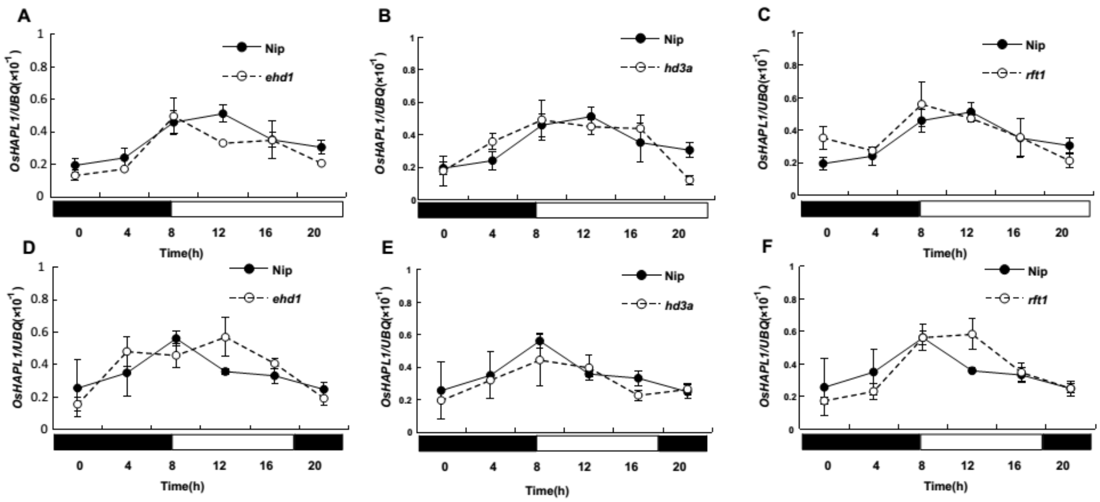

Fig. S5. Expression analysis of *OsHAPL1* between Nipponbare and *ehd1*, *hd3a*, and *rft1* mutants, under CLD (A-C) and CSD (D-F) conditions.

The plants were grown in growth chambers under LDs (14 h light /10 h darkness) and SDs (10 h light /14 h darkness) for 30 days. White bars indicate light; Black bars indicate darkness. The rice *Ubiquitin* (*UBQ*) gene was used as the internal control. Values represent means ± standard deviation from three independent biological replicates. X-axis: Time (h).

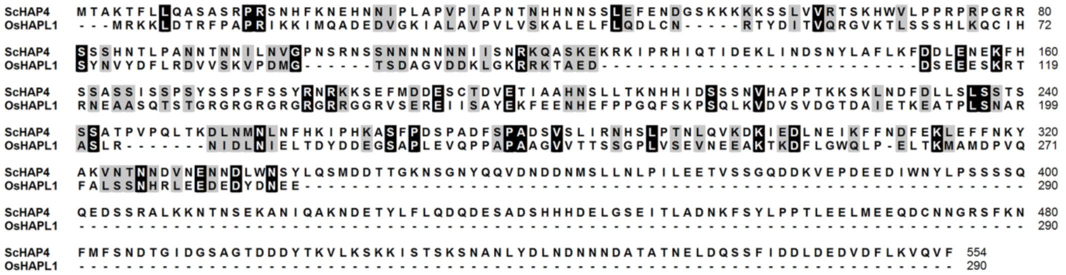

Fig. S6. Blast analysis of the amino acid sequences of OsHAPL1 and ScHAP4.

Table S1. Primers used in this study.

OsHAPL1-Flag-F	TCTGCACTAGGTACCTGCAGATGAGGAAGAAGCTGGACACC
OsHAPL1-Flag-R	ATGGATCCGTCGACCTGCAGTTCTTCGTTGTCATAATCCTC
PAN580-OsHAPL1-F	CGGTCCCGGGGGATCATGAGGAAGAAGCTGGACACCC
PAN580-OsHAPL1-R	TGCTCACCATGGATCGTTCTTCGTTGTCATAATCCTCA
ProGOsHAPL1-F	GGGCCCGGCGCGCCACAAATGCATCCTATGAATACC
ProGOsHAPL1-R	ATGGATCCGTCGACCGGTTCTTCGTTGTCATAATCCTC
CRISPR- OsHAPL1-F	AGATGATCCGTGGCAACACCCGCTTCCCCGCGGTAGTTTTAGAGCTATGC
CRISPR- OsHAPL1-R	GCATAGCTCTAAAACTACCGCGGGGAAGCGGGTGTTGCCACGGATCATCT
BD-OsHAPL1-F	CATGGAGGCCGAATTCATGAGGAAGAAGCTGGACACCC
BD-OsHAPL1-R	GGATCCCCGGGAATTCTTCTTCGTTGTCATAATCCTCA
AD-OsHAPL1-F	GGAGGCCAGTGAATTCATGAGGAAGAAGCTGGACACCC
AD-OsHAPL1-R	CGAGCTCGATGGATCCTTCTTCGTTGTCATAATCCTCA
BD-DTH8-F	CATGGAGGCCGAATTCATGAAGAGTAGGAAGAGCTATGGG
BD-DTH8-R	GGATCCCCGGGAATTCCTAATTAGTATTCCTCTTTAGTCTAAACC
AD-DTH8-F	GGAGGCCAGTGAATTCATGAAGAGTAGGAAGAGCTATGGG
AD-DTH8-R	CGAGCTCGATGGATCCCTAATTAGTATTCCTCTTTAGTCTAAACC
AD-DTH8H-F	GGAGGCCAGTGAATTCATGAAGAGTAGGAAGAGCTATGGG
AD-DTH8H-R	CCTGTCCTGCTCCTTCGCCTCGAACCCCAGCGTGGT
BD-OsHAPL1H-F	CATGGAGGCCGAATTCATGAGGAAGAAGCTGGACACC
BD-OsHAPL1H-R	GGATCCCCGGGAATTCGTCTGGAACTTTACTGACGACAT
GST-DTH8-F	CGCGTGGATCCCCGGAA ATGAAGAGTAGGAAGAGCTATGGG
GST-DTH8-R	GTCGACCCGGGAATTCATTAGTATTCCTCTTTAGTCTAAACC
MBP-OsHAPL1-F	AAGGATTTCAGAATTCATGAGGAAGAAGCTGGACACC
MBP-OsHAPL1-R	TAGAGGATCCGAATTTCATTCTTCGTTGTCATAATC
MBP-Hd1-F	AAGGATTTCAGAATTCATGAATTATAATTTTGGTGGCA
MBP-Hd1-R	TAGAGGATCCGAATTTCAGAACCATGGAACAGTACC
HIS-Hd1-F	TGGGTCGCGGATCCGAAATGAATTATAATTTTGGTGGCA
HIS-Hd1-R	GACGGAGCTCGAATTCTCAGAACCATGGAACAGTACC
BiFC-DTH8-F	CGATAGTACTGTCGACATGAAGAGTAGGAAGAGCTATGGG
BiFC-DTH8-R	TACCCTCGAGGTCGACATTAGTATTCCTCTTTAGTCTAAACC
BiFC-Hd1-F	CGATAGTACTGTCGACATGAATTATAATTTTGGTGGCA
BiFC-Hd1-R	TACCCTCGAGGTCGACGAACCATGGAACAGTACC
BiFC-OsHAPL1C-F	CGATAGTACTGTCGACATGAGGAAGAAGCTGGACACCC
BiFC-OsHAPL1C-R	TACCCTCGAGGTCGACTTCTTCGTTGTCATAATCCTCA
BiFC-OsHAPL1N-F	CGATAGTACTGTCGACATGAGGAAGAAGCTGGACACCC
BiFC-OsHAPL1N-R	TACCCTCGAGGTCGACTTCTTCGTTGTCATAATCCTCA
AD-OsHAP2AF	GGAGGCCAGTGAATTCATGAAGCCAGATGGTGAAACTC
AD-OsHAP2AR	CGAGCTCGATGGATCCTCATACAACATCGGACGCATC

AD-OsHAP2BF	GGAGGCCAGTGAATTCATGACGTCTGTAGTTCATGATGTTT
AD-OsHAP2BR	CGAGCTCGATGGATCCTTAGTTACTGGTTTTTGACTTCTTATTT
AD-OsHAP2CF	GGAGGCCAGTGAATTCATGCTCCCTCCTCATCTCACAG
AD-OsHAP2CR	CGAGCTCGATGGATCCAGCGCCCTCTTTCGCACGA
AD-OsHAP2DF	GGAGGCCAGTGAATTCATGGAGTCGAGGCCGGGG
AD-OsHAP2DR	CGAGCTCGATGGATCCTCATGTTTCCTTCTGTAGGAGCTGTAAG
AD-OsHAP2EF	GGAGGCCAGTGAATTCATGATAATGCTGTTGCAAGAAATG
AD-OsHAP2ER	CGAGCTCGATGGATCCCCTCATGACGGGGACACG
AD-OsHAP2FF	GGAGGCCAGTGAATTCATGATGAGCTTCAACAAGAGCCA
AD-OsHAP2FR	CGAGCTCGATGGATCCTTTGAGGAGGTCACAGCAGCC
AD-OsHAP2GF	GGAGGCCAGTGAATTCATGCTAATGCTTTTGCGACA
AD-OsHAP2GR	CGAGCTCGATGGATCCTCACCTCATCATGGAAACGC
AD-OsHAP2HF	GGAGGCCAGTGAATTCATGCTGAGCTTCAAGCAGAGCCA
AD-OsHAP2HR	CGAGCTCGATGGATCCACGCCTTGAGGAGCTCGCAG
AD-OsHAP2IF	GGAGGCCAGTGAATTCATGGCGCCATCGCTGCTC
AD-OsHAP2IR	CGAGCTCGATGGATCCCTAAATGGGAGACGTGGTGACG
AD-OsHAP2JF	GGAGGCCAGTGAATTCATGGGCCTACCTGAAAGGG
AD-OsHAP2JR	CGAGCTCGATGGATCCTCACACCTTGAGGAGGTCGA
AD-OsHAP3AF	GGAGGCCAGTGAATTCATGATGATGATGGATCTAGGGTTTT
AD-OsHAP3AR	CGAGCTCGATGGATCCATTCAATGCTAGGGTACTTGCAAT
AD-OsHAP3BF	GGAGGCCAGTGAATTCATGGCGGATGGGCCGGGGA
AD-OsHAP3BR	CGAGCTCGATGGATCCTCAGTTTGAGACATCCCCATTATGGTACTG
AD-OsHAP3CF	GGAGGCCAGTGAATTCATGTCGGAGGGGTTCGACG
AD-OsHAP3CR	CGAGCTCGATGGATCCCATGCCTTCAAACGATGATCCA
AD-OsHAP3DF	GGAGGCCAGTGAATTCATGGAGCCCGCATTTCCC
AD-OsHAP3DR	CGAGCTCGATGGATCCTTACTCATAGTTGGGGCCGACC
AD-OsHAP3EF	GGAGGCCAGTGAATTCATGGAGGCCGGCTACCCG
AD-OsHAP3ER	CGAGCTCGATGGATCCCTACTTGTATCCGAACGGATGCTG
AD-OsHAP3FF	GGAGGCCAGTGAATTCATGCCGGATTCGGACAACG
AD-OsHAP3FR	CGAGCTCGATGGATCCTCATAGCCTGTCTTGCCGG
AD-OsHAP3GF	GGAGGCCAGTGAATTCATGGCCGACCACCATGGC
AD-OsHAP3GR	CGAGCTCGATGGATCCGAACTGCCTGGAGCTATCCGTC
AD-OsHAP3HF	GGAGGCCAGTGAATTCATGAAGAGTAGGAAGAGCTATGGGC
AD-OsHAP3HR	CGAGCTCGATGGATCCTCATGCGTGGAGCCGGAG
AD-OsHAP3IF	GGAGGCCAGTGAATTCATGCCGGACTCGGACAACG
AD-OsHAP3IR	CGAGCTCGATGGATCCTCAAGCGCCCCTGTTTTGC
AD-OsHAP3JF	GGAGGCCAGTGAATTCATGAGCCGCGATCTCAATTTC
AD-OsHAP3JR	CGAGCTCGATGGATCCTTAATTGTTTGGACGACCTTGGTT
AD-OsHAP3KF	GGAGGCCAGTGAATTCATGGCAGGGAACAAAAAGCG
AD-OsHAP3KR	CGAGCTCGATGGATCCTCACATATTTTTTCCATAGCCATATCC
AD-OsHAP5AF	GGAGGCCAGTGAATTCATGGAACCATCCTCACAGCCTC
AD-OsHAP5AR	CGAGCTCGATGGATCCTTAGCTACTTTCTGGCAGAGAGTGC

AD-OsHAP5BF	GGAGGCCAGTGAATTCATGGAGCCATCATCACAACCT
AD-OsHAP5BR	CGAGCTCGATGGATCCTTAATCACTTTGCTGCTCTGC
AD-OsHAP5CF	GGAGGCCAGTGAATTCATGTCGAGGCTAGCGGCGG
AD-OsHAP5CR	CGAGCTCGATGGATCCCTATTCGGAGCTTGGAGGTGCA
AD-OsHAP5DF	GGAGGCCAGTGAATTCATGGAGCCCAAATCCACCA
AD-OsHAP5DR	CGAGCTCGATGGATCCCTGCGGCTGGACATAGTAGTAGG
AD-OsHAP5EF	GGAGGCCAGTGAATTCATGGATCCCACCAAATCCAG
AD-OsHAP5ER	CGAGCTCGATGGATCCCTGCTGGGGCACATAGTAGTAG
AD-OsHAP5FF	GGAGGCCAGTGAATTCATGGCCCGCACACCCGCC
AD-OsHAP5FR	CGAGCTCGATGGATCCTCATTCATCTTGTCCACCACCATCAG
AD-OsHAP5GF	GGAGGCCAGTGAATTCATGAGGCAGGCGAGGCCC
AD-OsHAP5GR	CGAGCTCGATGGATCCTTACTTATAGTCCGTATGATCGTCATCATG
AD-TFIIA-F	ATGGAGGCCAGTGAATTCATGGCCAGCAGCAACGTC
AD-TFIIA-R	CCCACCCGGGTGGAATTCTCAAAAATCAAATTCGCCGG
AD-TFIIB-F	ATGGAGGCCAGTGAATTCATGCACGACGCGGCG
AD-TFIIB-R	CCCACCCGGGTGGAATTCCTAGCCACCGAAGAGGACGC
AD-TFIID-F	ATGGAGGCCAGTGAATTCATGGATCCCCTCATAAAGCTC
AD-TFIID-R	CCCACCCGGGTGGAATTCCTATTTCGCAGATGATTCTGCTG
AD-TFIIF-F	ATGGAGGCCAGTGAATTCATGGGGAGCGCCGACC
AD-TFIIF-R	CCCACCCGGGTGGAATTCTCACTTCTTGTCATCTCTAAGGACG
AD-TFIIH-F	ATGGAGGCCAGTGAATTCATGGCCTCCTCCAAGCTCTAC
AD-TFIIH-R	CCCACCCGGGTGGAATTCCTACTGCCTTTGATCAGGTACAGAA

Table S2. Comparison of major agricultural traits between Kia-ake and *oshapl1* under natural long-day conditions.

Genotype	Plant height, cm	Tillers	Panicle length, cm	Primary branches n/panicle	Secondary branches n/panicle	Grains/panicle	1,000-grains weight, g	Yield per plant,g
Kita-ake	70.94± 2.21	20.58± 3.20	13.94 ± 0.71	6.64 ± 0.67	10.18± 1.33	70.45 ± 6.70	27.94± 1.19	26:37± 0.89
OsHAPL1-CRISPR-2	59.56± 2.83	21.47± 3.78	13.32 ± 0.80	6.00 ± 0.63	7.36 ± 1.80	53.27± 7.90	28.01± 0.91	21:21± 1.20
OsHAPL1-CRISPR-4	60.61± 2.26	20.18± 1.83	12.70 ± 0.46	6.58 ± 0.51	7.67± 0.79	54.17± 6.64	27.22± 0.75	19.21± 0.70

Statistics of agronomic traits are based on kita-ake, and two independent OsHAPL1-CRISPR transgenic plants under NLD. Agronomic traits are means ± standard deviations (n = 20).
